# Field testing a new ICD coding system: methods and early experiences with ICD-11 Beta Version 2018

**DOI:** 10.1186/s13104-022-06238-2

**Published:** 2022-11-08

**Authors:** Cathy A. Eastwood, Danielle A. Southern, Shahreen Khair, Chelsea Doktorchik, Denise Cullen, William A. Ghali, Hude Quan

**Affiliations:** 1 Department of Community Health Sciences, Cumming School of Medicine, University of Calgary, 3280 Hospital Drive NW, Calgary, AB T2N 4Z6 Canada; 2 O’Brien Institute for Public Health, Cumming School of Medicine, University of Calgary, 3280 Hospital Drive NW, Calgary, AB T2N 4Z6 Canada; 3 2431 22A Street NW, Calgary, AB T2M 3X8 Canada; 4 University of Calgary, 2500 University Drive NW, Calgary, AB T2N 1N4 Canada

**Keywords:** Dually-coded database, ICD-10-CA, ICD-11, Chart review, Inter-rater reliability, Data, ICD-11 field trial, ICD-11 reference guide

## Abstract

**Objective:**

A beta version (2018) of International Classification of Diseases, 11th Revision for MMS (ICD-11), needed testing. Field-testing involves real-world application of the new codes to examine usability. We describe creating a dataset and characterizing the usability of ICD-11 code set by coders. We compare ICD-11 against ICD-10-CA (Canadian modification) and a reference standard dataset of diagnoses. Real-world usability encompasses code selection and time to code a complete inpatient chart using ICD-11 compared with ICD-10-CA.

**Methods and results:**

A random sample of inpatient records previously coded using ICD-10-CA was selected from hospitals in Calgary, Alberta (N = 2896). Nurses examined these charts for conditions and healthcare-related harms. Clinical coders re-coded the same charts using ICD-11 codes. Inter-rater reliability (IRR) and coding time improved with ICD-11 coding experience (23.6 to 9.9 min average per chart). Code structure comparisons and challenges encountered are described. Overall, 86.3% of main condition codes matched. Coder comments regarding duplicate codes, missing codes, code finding issues enabled improvements to the ICD-11 Browser, Coding Tool, and Reference Guide. Training is essential for solid IRR with 17,000 diagnostic categories in the new ICD-11. As countries transition to ICD-11, our coding experiences and methods can inform users for implementation or field testing.

**Supplementary Information:**

The online version contains supplementary material available at 10.1186/s13104-022-06238-2.

## Introduction

Coded health data are important for health services funding, physician payment, and research [1]. World Health Organization (WHO) encouraged testing International Classification of Diseases for Mortality and Morbidity Statistics, 11th Revision (ICD-11 Beta Version 2018) before release in 2018, and transition from the previous version of ICD.

New features include: (1) code-clustering; (2) new extension code chapter for disease severity, progression, and timing; (3) digital ICD-11 Browser and Coding Tool for code searching [1, 2]. ICD-11codes are alphanumeric, with first character indicating chapter and a number at the third character position (1A00.00 to ZZ9Z.ZZ). ICD-11 contains 5 new chapters and over 17,000 diagnostic categories and over 100,000 medical index terms allowing for a greater description of health conditions [3, 4]. ICD-11 enables adding detail to coded entities using several mechanisms. Healthcare-related harms coding in ICD-11 involves cluster coding of injury, cause, and mode, known as the 3-part model [5].

ICD versions differ in their number of codes, chapters, and subcategories. Specific diagnosis codes are present in some but not all modifications [6]. A dually-coded database is required to compare ICD version similarities and differences in code usage between systems. A database with reference standard labelled records is essential to quantify this comparison. [6].

We conducted a large field trial to further ICD-11 development through real-world coding. The objective was to create a 3-part data set and test usability of the ICD-11 code set (Beta version 2018) compared to ICD-10-CA code set by professional coders in an inpatient setting. Real-world usability encompasses code selection and time to code complete inpatient charts using ICD-11 compared with ICD-10-CA. As countries begin transitioning to ICD-11, our coding experiences and methods can inform users for implementation or field testing.

## Materials and methods

**Fig. 1:**
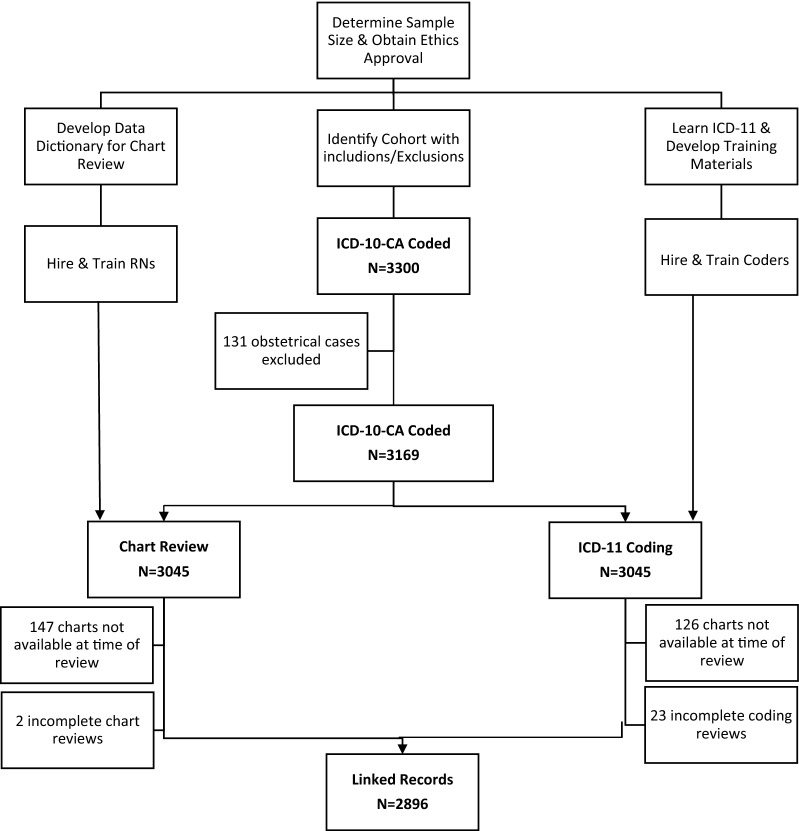
Steps for creating a chart review reference dataset, and a dually-coded dataset

We generated and linked three data sets: (1) a retrospective clinical chart review as reference standard; (2) original ICD-10-CA coded data; (3) re-coded ICD-11 coded data (Fig. 1). We compared ICD-11 codes against ICD-10-CA (a Canadian modification of International Classification of Diseases, Tenth Revision) codes [7], and a reference standard data set of diagnoses.

### Sample size and cohort

With cost and time in mind, 1,000 charts from 3 hospitals were selected for review. Sample size was based on previous [8] findings on sensitivity and prevalence of conditions in a sample of ICD-10-CA data. A sample of 3000 records was required to test a large enough difference in sensitivity (10%) for common conditions (e.g., myocardial infarction (12.8%), cardiac arrhythmia (21.8%), hypertension (30.2%)). We determined that ten percent was large enough to detect a difference without coding changes [8].

Random discharge charts were selected from records between January-June 2015, from three major diverse teaching hospitals (577–1100 beds) in Calgary, Alberta. Patients were 18 and 104 years old with an Alberta Personal Health Number. Psychiatric admissions were included. Obstetric admissions were excluded. We selected the first 1100 records from each hospital. The additional 100 records per site allowed for missing or excluded charts.

### Chart review dataset

Internal validation of a dually-coded database involves measuring how well codes, selected from ICD-10-CA and ICD-11, compare with each other, and align with the conditions identified by chart reviewers [8]. Estimates for sensitivity, specificity, positive and negative predictive values can be estimated.

### Data dictionary

We replicated the chart review approach from our prior study on validity of ICD-10-CA [8]. We selected 51 medical conditions, including Charlson and Elixhauser [8–11] conditions commonly used for risk adjustment, and up to 3 harms (Additional file 1). Harms included healthcare-related adverse events (injury, illness, disability, or death arising in hospital), specifying harm, cause, and mode. Definitions were based on literature [9, 10] and our prior validation study [8]. Where no published definition was available, ICD-11 Browser definitions (beta version) were used [2]. Condition definitions, including a list of potential harms, are available in the Data Dictionary for ICD-11 Field Trial (see Additional file 2).

### Chart review team

Research coordinator (CE) trained 6 nurse chart reviewers. Training involved learning condition definitions and following a consistent order to review chart documents. Nurse reviewers examined entire charts for specific health conditions and were blinded to ICD codes assigned by coders.

### ICD-10-CA coded dataset

The existing ICD-10-CA dataset represented “real-life” coding. Alberta hospitals employ trained clinical coders (CCs) (i.e., nationally certified health information management specialists) who read through patient hospital charts. These CCs assigned ICD-10-CA codes to describe patients’ diagnoses, based on ICD-10-CA Canadian coding standards [12]. Each discharge record contains a unique identification number and up to 25 fields for diagnosis codes, which became the study dataset. Procedure codes (typically coded using Canadian Classification of Health Interventions) were excluded from chart review and re-coding for time efficiency and cost.

### Re-coded ICD-11 dataset

Phase 3 involved re-coding the same inpatient charts using ICD-11.

### Training materials

Research coordinator (CE) and employee of Canadian Institute for Health Information (CIHI) (DC) developed ICD-11 training materials [14] to augment WHO Education and Implementation Committee (EIC) information sheets [20]. We developed slide sets covering ICD-11 concepts and tools [13, 14]. Coding practice materials included two sets of Morbidity and Quality and Safety Case Scenarios. We developed coding rules and decision trees for coding hospital-acquired conditions (harms) with the WHO Quality and Safety Technical Advisory Group.

### Clinical coding team

We (researchers, CIHI, and a WHO coding consultant) trained 6 CCs in ICD-11 concepts. Training involved 20 classroom hours and 40 hours of coding practice homework before coding complete hospital charts. The coding team and trainers met monthly during coding phase to discuss coding issues. ICD-11 coding decisions were based on what was available at the time in the draft ICD-11 Reference Guide of the WHO [15], WHO ICD-11 Coding Tool [16], and Canadian ICD-10-CA coding standards [12], given that ICD-11 coding rules were limited. CCs were blinded to ICD-10-CA codes and chart review information. The coding team was encouraged to use the Coding Tool first, then use the Browser if needed. The Coding Tool offered the ability to search by word-matching, including synonyms, and quick visual reference to possible codes [4]. The Browser required more specific searching by body system and scanning the hierarchical lists for code options.

## Analysis

### Test inter-rater reliability (IRR) of chart review

IRR involved 2 nurses reviewing sets of the same 10 charts for agreement on the presence of 17 Charlson conditions. Where agreement was poor (kappa < 0.60), retraining took place and chart review resumed in batches of 10 charts, until high agreement (kappa > 0.8) [17]. IRR was not available for the ICD-10-CA dataset.

### Test IRR of ICD-11 coded charts

IRR involved 60 charts coded by 2 CCs. IRR focused on consistent coding of the main condition given the bulk of possible codes generated from complete hospital charts. After 40 charts, kappa of 0.50 was reached on the main condition parent code, meaning the highest level in the ICD-11 condition hierarchy, (e.g., BA41myocardial Infarction vs BA41.0 Acute ST elevation myocardial infarction). Training continued, differences were discussed, experts were engaged for guidance until high agreement was achieved (> 0.8), and independent coding proceeded.

## Results

Results include test IRR results, final database, coding time by location and chart complexity, ICD-10 and ICD-11 coding comparison examples, and coding challenges.

### Test IRR

For chart review, high agreement (kappa > 0.80) for condition detection was reached after 2 people completed 49 sets of charts. For ICD-11 coding, kappa of 0.88 was reached for main condition parent codes after coding a total of 60 charts.

### Sample

The sample started with 3300 charts coded in ICD-10-CA. A sequential list of these charts was selected for each task (chart review and ICD-11 coding) for 3045 charts and was combined. Unavailable charts were skipped, and the next chart was selected. Chart review and ICD-11 coding were done at different times and chart availability differed. The final sample for the dually-coded database, was n = 2896 (Fig. 1).

### Time and hospital record characteristics

**Fig. 2:**
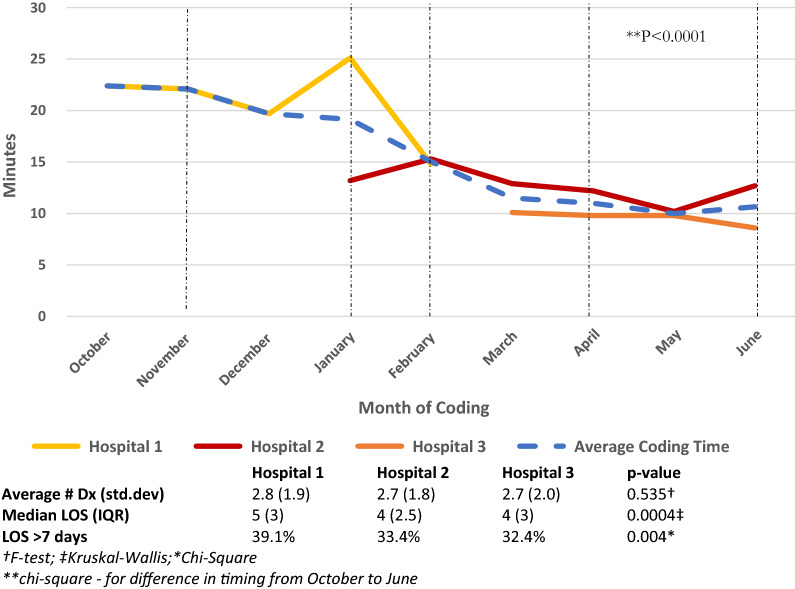
ICD-11 coding time by hospital over time

Figure 2 presents ICD-11 coding time by hospital over nine months. Chart review time averaged 14.6 min (std. dev. 29.1, median 11.0 min). Given that the charts had been previously coded with ICD-10-CA, no specific time was measured per chart. However, the average time to code an acute care hospital chart using ICD-10-CA in Calgary is approximately 15–20 min given that the productivity expectation measure is 15.6 min [18]. ICD-11 Coding time declined from 23.6 (std. dev. 14.1) to 9.9 min (std. dev. 6.4) on average per chart (p < 0.0001), as coding proficiency increased over time (Fig. 2). Coding time in Hospital #1 was related to two factors – learning the new coding system, and chart length. Hospitals were coded sequentially and both Hospitals 2 and 3 tended to have fewer complex charts due to less acute patients. Length of stay (LOS) in days was longer in Hospital #1 compared with #2 and #3 (median LOS (IQR) = 5(3), 4(2.5), 4(3), respectively p = 0.0004). Chart complexity (number of diagnoses coded) did not differ significantly between hospitals (p = 0.535).

### Code structure comparison

ICD-11 code structure includes stem codes (main diagnosis or symptom code) with clustering and extension coding for detailed descriptions of conditions. ICD-10 contains greater precoordinated terms, while ICD-11 enables codes to be postcoordinated and clustered. Code structure comparison examples are in Additional file 3. Several diagnoses that required multiple codes in ICD-10-CA are now described in single code clusters in ICD-11, such as healthcare-related harms.

### Main condition comparison

An analysis of 2018 main conditions (as defined by ICD-10-CA) compared ICD-10-CA and ICD-11. We found that overall, 86.3% of main condition coding matched (Table 1). Examples of partial matches are included in Table 1 as codes that were more specific in one coding system but identified the same concept.

**Table 1:** Main condition code matches between ICD-10-CA and ICD-11

Matches	N	%
Complete	1403	69.5%
Partial	339	16.8%
No	276	13.7%
Total	2018	100

Examples of partial main condition code matches	ICD 11 code	ICD 10-CA code
Example 1	GB70.1-Calculus of ureter	N20.0-Calculus of kidney
Example 2	GB56.0-Hydronephrosis with uteropelvic junction obstruction	N13.5-Kinking and stricture of ureter without hydronephrosis
Example 3	2C82.0-Adenocarcinoma of prostate	C61-Malignant neoplasm of prostate
Example 4	DA42.Y-Other specified gastritis	A04.3-Enterohaemorrhagic *Escherichia coli* infection

### Healthcare-related harms

Early analysis of Hospital #1 records (n = 1009) indicated healthcare-related harms were coded using ICD-11 in 88 records (8.7%) [19]. Compared to chart review, sensitivity and specificity were 31.3% and 94.6% respectively. ICD-11 had NPV (45.5%) and PPV (90.5%) compared to ICD-10.

### ICD-11 coding challenges

Challenges with IRR using the new ICD-11 classification system were multifactorial. Comprehensive ICD-11 contains 17,000 unique codes [20], thus, more code choices, while ICD-10-CA contains only 12,420 codes [21]. CCs required training for new code structures like code clustering [22] (Additional file 3). Also, as evidenced by low specificity in Hospital #1 coding [19, 23, 24], coding harms in ICD-11 was particularly challenging. Coders robustly discussed code selection for complex cases and harms.

Circumstances during data collection, like new codes and coding procedures for ICD-11 being under revision, made training and learning challenging. Training CCs on complex case scenarios required the most time. New ICD-11 training materials are now available from the WHO [25] and the EIC committee [26], and the ICD-11 Browser, Reference Guide, and Coding Tool are refined [2].

## Discussion

These methods are available for other countries testing and adopting ICD-11. Usability was demonstrated with similar time to code and consistently high levels of main condition code matches when compared to either ICD-10-CA coding, or healthcare harms and chart review. Coding complete charts with ICD-11enabled refinement of the new classification system for all stakeholders to benefit. Greater code detail is possible without adding coding time.

Previous studies discuss similar advantages and challenges related to understanding and identifying the 3-part model for coding healthcare-related harms [23, 24]. This paper demonstrates the differences in complexity when coding main condition and more complex situations like healthcare-related harms (Additional file 3).

### Recommendations made for the ICD-11 reference guide and ICD-11

Our study enabled feedback to the WHO on the new ICD-11 codes, coding tools, and Reference Guide. Many changes were integrated into these tools and ICD-11 Browser prior to release, by the WHO consultant or via proposals to the WHO from advisory groups. Changes to the ICD-11 Reference Guide for the morbidity-related chapters included improved clinical definitions, and expanded cluster coding and postcoordination [15]. Substantial content was added to clarify Chapter 23, External Causes of Morbidity and Mortality. ICD-11 Reference Guide now includes a framework and guidelines for using the three-part model to code healthcare-related harms [15]. ICD-11 improvements included resolving missing codes and inclusion terms, postcoordination linkages, substance/medication list, 3-part model coding, and functions of the Coding Tool. Examples of ICD-11 changes are listed in a Additional file 4.

## Conclusion

This paper describes real-world usability of the 2018 Beta Version of the ICD-11 code set by professional CCs and challenges in an inpatient setting. Coders selected ICD-11 codes and coded complete acute care records in a timely manner. Length of stay contributed to longer coding times. Training was crucial for strong IRR for a new classification system. The study was timely and provided recommendations for ICD-11 enhancement prior to its public release. Overall ICD-11 was well received by coders and a high degree of matches were achieved for main condition codes. As countries begin transitioning to ICD-11, these methods can be replicated for field testing and inform users for implementation worldwide.

## Limitations

Several limitations exist. First, this study was performed at 3 hospitals in one city. Usability of ICD-11 for mortality coding or morbidity coding in outpatient settings, home care settings, or low-resource settings is unknown. Second, while ICD-10-CA codes were collected in “real-life” settings with various CCs, ICD-11 codes were collected in controlled research setting with six trained CCs. Third, CCs did not code the same charts with both ICD-10-CA and ICD-11. It is possible, in their prior coding roles, that they may have coded the same charts. None of the coders remembered coding the same charts with ICD-10-CA. Fourth, definitions for each harm were not provided to the teams which may account for the reduced sensitivity in Hospital #1 [24]. CCs relied on ICD-10-CA coding rules and chart reviewers relied on clinical knowledge. Lastly, this study occurred when ICD-11 was developing and changing. To achieve moderate coding agreement, we chose the parent-level rather than the code-specific level to compare. Focusing on clinical conditions for chart review, we looked at prevalence of high-level categories rather than specific diagnoses. To our knowledge this is the first direct comparison of ICD-10 and ICD-11 in a dually-coded database. The large sample included a wide variety of conditions to be coded enabling good validity precision to be achieved.

## Supplementary Information

**Additional file 1.** Chart review conditions for data collection

**Additional file 2.** Data Dictionary for ICD-11 Field Trial

**Additional file 3.** Comparison between ICD-10-CA and ICD-11 Coding Structure

**Additional file 4.** Examples of changes made to ICD-11

## Data Availability

Due to data sharing policies and the Data Disclosure Agreement of the data custodians, the dataset is not able to be made publicly available. It may be able to be shared only to researchers in Alberta with approval from the data custodians. (https://www.albertahealthservices.ca/research/Page16074.aspx).

## Abbreviations

ICD: International Classification of Diseases
ICD-11: International Classification of Diseases, 11th revision for Mortality and Morbidity Statistics
ICD-10-CA: Canadian specific version of the international classification of diseases, tenth revision
CC: Clinical coders
WHO: World Health Organization
EHR: Electronic health record
WHO-CC: World Health Organization collaborating centre
CHREB: Conjoint Health Research Ethics Board
PHN: Personal health number
SCM: Sunrise Clinical Manager™
IRR: Inter-rater reliability
CIHI: Canadian Institute for Health Information
EIC: Education and Implementation Committee
