# Supplementary material for: Field testing a new ICD coding system: methods and early experiences with ICD-11 Beta Version 2018
Source: BMC Res Notes. 2022 Nov 8;15:343. doi: 10.1186/s13104-022-06238-2 (PMC9644463; doi:10.1186/s13104-022-06238-2)
Supplement: Supplementary file 1 — Additional file 1. Chart review conditions for data collection [file 13104_2022_6238_MOESM1_ESM.docx]

**Additional file 1** Chart review conditions for data collection

| **Conditions** | | | |
| --- | --- | --- | --- |
| 1. Angina | 18. Inflammatory bowel disease | 35. HIV/AIDS |  |
| 2. Myocardial infarction (new) | 19. Liver disease | 36. Disorders due to tobacco use |  |
| 3. Myocardial infarction (old) | 20. Cancer | 37. Dyslipidemia |  |
| 4. Congestive heart failure | 21. Malignancy without metastases | 38. Disorders due to alcohol use |  |
| 5. Cardiac arrhythmias | 22. Malignancy with metastases | 39. Disorders due to drug use |  |
| 6. Atrial fibrillation | 23. Leukemia | 40. Psychoses |  |
| 7. Atrial flutter | 24. Lymphoma | 41. Anxiety |  |
| 8. Valve disease | 25. Renal disease | 42. Depression |  |
| 9. Pulmonary circulatory disorders | 26. Rheumatologic disease | 43. Homeless |  |
| 10. Hypertension | 27. Diabetes | 44. Urinary tract infection |  |
| 11. Peripheral vascular disease | 28. Hypothyroidism | 45. Pneumonia |  |
| 12. Cerebrovascular disease | 29. Coagulopathy | 46. Skin/wound infection |  |
| 13. Paralysis | 30. Anemia | 47. Gastroenteritis |  |
| 14.Chronic pulmonary disease | 31. Fluid & electrolyte disorder | 48. Other infection |  |
| 15. Asthma | 32. Obesity | 49. Sepsis |  |
| 16. Peptic ulcer Disease | 33. Significant weight loss | 50. Pressure Ulcer |  |
| 17. Gastrointestinal bleed | 34. Dementia | 51. Sleep disorders |  |
| **Harms** (capture up 3 of the first occurring healthcare-related harms) | | | |
| Harm 1 | Harm 2 | Harm 3 |  |
