# Supplementary material for: Field testing a new ICD coding system: methods and early experiences with ICD-11 Beta Version 2018
Source: BMC Res Notes. 2022 Nov 8;15:343. doi: 10.1186/s13104-022-06238-2 (PMC9644463; doi:10.1186/s13104-022-06238-2)
Supplement: Supplementary file 2 — Additional file 2. Data Dictionary for ICD-11 Field Trial [file 13104_2022_6238_MOESM2_ESM.docx]

**Additional file 2**

**DATA DICTIONARY FOR ICD-11 FIELD TRIAL**

**DEMOGRAPHIC DATA**

1. Transfer in from another acute care hospital? Y/N
   1. Use face sheet, ED notes, history & physical
2. Did the patient have an inpatient visit within 1 year before this admission? Y/N
3. Note the discharge date of the most recent inpatient visit within 1 year before this admission.
4. **Discharge disposition**. Refer to Discharge Summary, Transitional Care Nurse note, Multidisciplinary Progress Notes, Orders, or Social Worker note. In this order.

01 – Transferred to another facility providing inpatient hospital care (includes other acute, sub-acute, psychiatric, rehabilitation, cancer centre/agency, pediatric hospital…)

02 – Transferred to a long term care facility (personal care homes, auxiliary care, nursing homes, extended care, homes for the aged, senior’s homes, veteran’s homes…)

03 – Transferred to other (palliative care/hospice, addiction treatment centre….)

04 – Discharged to a home setting with support services (senior’s lodge, attendant care, home care, meals on wheels, homemaking, supportive housing….)

05 – Discharged home

06 – Signed out (against medical advice)

07 – Died

1. **Admission weight (kg) (**closest to admit date) First occurring in Sunrise Clinical Manager (SCM), then Nursing Admission
2. **Admission height (cm) (**closest to admit date) First occurring in SCM, then Nursing Admission

**CONDITIONS** (World Health Organization, 2015, 2016)

Definitions for most conditions listed came from ICD-11 or ICD-10 classification websites, where no other clinical definitions existed in the literature.

**Condition Present – Yes/No =** must be listed as a diagnosis in the History & Physical, Multidisciplinary Progress notes, Consult notes, or Discharge Summary

**Timing of Condition**

**Present on admission** includes ‘History of’ and present on day of admission. Also includes chronic conditions documented later during the admission.

**Developed after admission** includes a new diagnosis or condition that occurred during the admission.

**Admit Category**

**Initial reason for admission** includes the first listed diagnosis on the History & Physical or if not found, the Emergency medical notes. Assign this to only one condition.

**Primary discharge** **diagnosis** includes the first listed diagnosis on the Discharge Summary. Assign to only one condition.

**Neither**

**Both**

1. **Angina** includes all forms of angina (e.g. stable, unstable, coronary vasospastic angina, microvascular angina, ischemic heart disease with angina). Includes acute coronary syndrome as a less specific term for ischemic heart disease. Excludes Chest pain for other reasons than diagnosis of angina (World Health Organization, 2015).
2. **Myocardial infarction (new)**- this admission includes patients with one or more definite or probable myocardial infarctions; had electrocardiographic and/or enzyme changes and myocardial necrosis. Patients with electrocardiographic changes alone are not designated as having had an infarction. ST Elevation MI (STEMI) and Non- ST Elevation MI (NSTEMI) are both designated as MI. Excludes acute coronary syndrome (angina) (World Health Organization, 2015).
3. **Myocardial infarction (old)**- Healed myocardial infarction; Past myocardial infarction diagnosed by ECG or other special investigation, but currently presenting no symptoms; comorbid condition (World Health Organization, 2015).
4. **Congestive heart failure** includes acute and chronic systolic or diastolic heart failure; includes left, right, and biventricular heart failure with reduced or preserved ejection fraction. Includes HF from congenital deformities, valvular disease, hypertension, or pregnancy; includes pulmonary edema with heart failure; includes cardiomyopathy (any kind); cardiomegaly if HF is also listed; if pulmonary hypertension, also look for right heart failure. Various forms of edema or anasarca can be due to HF; so can portal hypertension and chronic or end-stage kidney disease (World Health Organization, 2015). *Can include post-procedural pulmonary edema or HF but then it must be designated as ‘developed after admission’.
5. **Cardiac arrhythmias** includes patients with sick sinus syndrome, ventricular arrhythmias requiring chronic treatment, like SVT (supraventricular tachycardia). Any of a large and heterogeneous group of conditions in which there is abnormal electrical activity in the heart. The heartbeat may be too fast or too slow, and may be regular or irregular (World Health Organization, 2015). Includes presence of a pacemaker or defibrillator, and AV Blocks or sudden cardiac death episodes. **EXCLUDES** atrial fibrillation and atrial flutter. Mutually exclusive categories.
6. **Atrial** **fibrillation**– includes chronic, intermittent, or paroxysmal atrial fibrillation (World Health Organization, 2015) not included in Cardiac Arrhythmias. Mutually exclusive.
7. **Atrial flutter** includes chronic, typical, or atypical atrial flutter (World Health Organization, 2015). Not included in Cardiac Arrhythmias. Mutually exclusive.
8. **Valve disease** includes any disease involving one or more heart valves (aortic, mitral, pulmonary or tricuspid). Rheumatic heart or valve disease is included. For example, patients with aortic stenosis and/or insufficiency, those with significant mitral stenosis and/or insufficiency, and those with prosthetic aortic or mitral valves and those with symptomatic mitral valve prolapse, tricuspid insufficiency. Includes abscess. Include presence of thrombosis, or rupture of heart valves; and artificial valves (World Health Organization, 2015).
9. **Pulmonary circulatory disorders** Includes enlargement of the right ventricle of the heart as a response to increased resistance or high blood pressure in the lungs (e.g. pulmonary hypertension, cor pulmonale), and diseases of pulmonary circulation (e.g. pulmonary embolism, ruptured pulmonary artery; acquired pulmonary artery or vein abnormality) (World Health Organization, 2015).
10. **Hypertension** includes patients with any form of hypertension diagnosis, including controlled hypertensives; essential hypertension (e.g. from renal artery stenosis; aging), secondary hypertension, or hypertensive crisis (World Health Organization, 2015).
11. **Peripheral vascular** **disease** includes diseases of arteries and arterioles with intermittent claudication or those who had a bypass for arterial insufficiency, those with gangrene or acute arterial insufficiency, and those with treated or untreated thoracic or abdominal aneurysm, or grafts to femoral or other peripheral arteries. Refers to obstruction of large arteries not within the coronary, aortic arch, or brain (World Health Organization, 2015).
12. **Cerebrovascular disease** includes a group of brain dysfunctions related to disease of the blood vessels supplying the brain, where hypertension is the most important cause. Hypertension should be mentioned. Includes a current or history of a cerebrovascular accident (stroke) with minor or no residual effects and transient ischemic attacks (TIA). Can include hemorrhagic stroke, ischemic stroke, or transient cerebral ischemia (intracranial and subarachnoid hemorrhages, cerebral infarctions). Includes aneurysm or dissection of the carotid artery; incudes carotid stenosis; carotid atherosclerosis. Excludes traumatic intracranial hemorrhage and vascular dementia (World Health Organization, 2015).
13. **Paralysis** includes patients with hemiplegia or paraplegia, whether it occurred as a result of a cerebrovascular accident or other condition. Incudes quadriplegia, monoplegia, and displegia (World Health Organization, 2015).
14. **Chronic pulmonary disease** includes COPD, chronic bronchitis, chronic bronchiectasis, emphysema, lung diseases from toxic exposures, chronic respiratory conditions due to exposure to gasses and fumes, from radiation, drug-induced chronic lung disease, & cystic fibrosis (World Health Organization, 2015). Excludes pulmonary heart disease (belongs under pulmonary circulation disorders).
15. **Asthma -** Acute or chronic inflammatory disorder of the airways in which many cells and cellular elements play a role. Includes episodes of wheezing, breathlessness, chest tightness, and coughing, particularly at night or in the early morning. Can include allergic or non-allergic asthma, and other asthmas induced by aspirin, exercise, cough/cold, croup (World Health Organization, 2015). (Include patients with asthma medications/ inhalers documented at admission and at discharge).

| **Combination Medications** | **Corticosteroids** | **Long-Acting Bronchodilators** |
| --- | --- | --- |
| Symbicort® | Budesonide (Pumicort®) | Formoterol (Foradil®, Oxeze®) |
| Advair® | Fluticasone (Flovent®) | Salmeterol (Severent®) |
|  | Monotasone (Asmanex®) |  |

| **Rescue Bronchodilators** | **Corticosteroid pills** |
| --- | --- |
| Fenoterol (Berotec®) | Prednisone |
| Formoterol (Foradil®, Oxeze®) | Prednisolone (PediaPred®) |
| Ipratropium (Atrovent®) | Dexamethasone (Decadrone®) |
| Isoproterenol (Isuprel®) |  |
| Orciprenaline (Alupent®) |  |
| Salbutamol (Ventolin® HFA, Apo-Salvent® CFC Free, Ratio-Salbutamol HFA) |  |
| Terbutaline (Bricanyl®) |  |

1. **Peptic ulcer disease** includes patients who have required treatment for ulcer disease, including those who have bled from ulcers. Includes GERD (gastric esophageal reflux disorder), gastric, duodenal, peptic, and gastrojejunal ulcers without hemorrhage or perforation. Excludes GI bleeds; acute hemorrhagic erosive gastritis, malignant neoplasms of stomach or intestines (World Health Organization, 2015). Include patients with acid-reducing medications documented at admission AND at discharge OR explicit diagnosis of peptic ulcer disease as listed above (group consensus).

**Drugs include**:

**Proton Pump Inhibitors:** Omeprazole (Prilosec®, Prilosec OTC®), Omeprazole magnesium (Losec), Lansoprazole (Prevacid®, Prevacid FasTab®), Rabeprazole, Rabeprazole sodium (Pariet®), Pantoprazole (Pantoloc®), Esomeprazole (Nexium®)

**Antacids:** Aluminum hydroxide (Almagel®, Maalox®, Mylanta®), Calcium carbonate (Tums®, Rolaids® Antacid®, Extra Strength Calcium Antacid Chewable®, Antacid 2®, Gastrocalm®, Bismuth®), Magnesium carbonate (Magmix®), Magnesium hydroxide (Milk of Magnesia®), Magnesium oxide, Magnesium trisilicate (Gasulsol®), Magnesium citrate (Citro-mag®), Calmax, Maalox, Sodium bicarbonate, Sodium citrate (Alka-Seltzer®, Bromo Madelon®, E-Z-Gas 2®)

**H2 Antagonists:** Cimetidine, Ranitidine (Zantac®), Famotidine (Pepcid®, Pepcid AC®), Nizatidine (Axid®)

1. **Gastrointestinal** **bleed** includes a group of signs and symptoms indicating bleeding into the digestive tract. This mainly refers to haemorrhage from organs in the gastrointestinal tract, but it can also occur from the biliary tract. These categories can be also used for post coordination codes as complications of underlying illness. Digestive system haemorrhage includes melena, hematemesis, hematochezia (fresh blood from anus), positive occult blood in stool, obscure GI bleeding (no endoscopic cause found) (World Health Organization, 2015).
2. **Inflammatory bowel disease** is a group of inflammatory conditions of the intestine of unknown etiology. The pathogenesis is hypothesized that the mucosal immune system shows an aberrant response towards luminal antigens such as dietary factors and commensal microbiota in genetically susceptible individuals. Includes patients with Crohn’s, ulcerative colitis, or indeterminate colitis (World Health Organization, 2015). Does NOT include diverticulits/osis.
3. **Liver disease** includes patients with metabolic liver disease, infectious liver disease, alcoholic liver disease (cirrhosis), portal hypertension and a history of variceal bleeding, all hepatitis acute or chronic. Includes hepatic failure, non-alcoholic fatty liver disease, drug/ toxin induced liver disease, autoimmune liver disease, polycystic liver disease, vascular disorders of the liver (World Health Organization, 2015).
4. **Cancer** includes any neoplasm (cancer) benign, metastatic, solid tumor, blood based, past, present. If yes, diagnosis will be one of the four categories below, or if none chosen = other.
5. **Malignant tumor without metastases** consists of patients with solid tumors without documented metastases, but initially treated in the last five years, including breast, colon, lung, and a variety of other tumors. If timing is not identified, still include (Quan et al., 2005).
6. **Malignant solid tumor with metastases- Metastatic cancer** includes patients with metastatic solid tumors, including breast, lung, colon, and other tumors (Quan et al., 2005).
7. **Leukemia** includes patients with acute and chronic myelogenous leukemia, acute and chronic lymphocytic leukemia, and polycythemia vera (Quan et al., 2005).
8. **Lymphoma** includes patients with Hodgkin and non-Hodgkin lymphomas (World Health Organization, 2015).
9. **Renal disease** includes renal tubule-interstitial diseases, that is, any disease characterized by pathological changes to the renal tubules and interstitial tissues.
   1. **Acute renal failure** including acute nephritis, pyelonephritis, acute tubular necrosis, acute renal infection (World Health Organization, 2015).
   2. **Chronic kidney disease** includes GFR <60 or presence of kidney damage that is present for more than 3 months. Evidence of kidney damage can include structural abnormalities (imaging or histology), albuminuria above normal limits, urinary sediment abnormalities or electrolyte disturbances due to tubular disorders. Includes chronic renal failure, chronic uremia, and chronic renal insufficiency. Includes patients on or off all forms of long-term dialysis (World Health Organization, 2015).
10. **Rheumatologic disease** includes patients with moderate to severe rheumatoid arthritis or bursitis, diseases with positive rheumatoid factor. Includes rheumatoid lung disease and vasculitis. Extra-articular features include nodules, pericarditis, pulmonary fibrosis, peripheral neuropathy, and amyloidosis. Can include heart involvement from rheumatic fever. Includes Fibromyalgia which is characterized by chronic diffuse pain, intense fatigue and sleep disturbances often associated with anxiety or depression, and triggered by physical or psychological trauma (World Health Organization, 2015).
11. **Diabetes – includes uncomplicated (Type 1 or 2)-** Also considered mild diabetes and includes all diabetes treated with insulin or oral hypoglycemics, but not diet alone; without end-organ damage or extreme blood glucose reactions. **And Diabetes, complicated** includes retinopathy, neuropathy nephropathy or circulatory disorders (if linked to diabetes). Includes patients who had previous hospitalizations for ketoacidosis, hyperosmolar coma, or control and those with juvenile onset or brittle diabetics (World Health Organization, 2015). Does not include ‘borderline diabetic’.
12. **Hypothyroidism** includes congenital (rare) or acquired (common) hypothyroidism; includes myxedema. Also includes subclinical iodine-deficiency hypothyroidism (World Health Organization, 2015).
13. **Coagulopathy** includes all coagulopathies including congenital (e.g. hemophilia) or acquired coagulation defects (e.g. DIC- disseminated intravascular coagulation, post-partum coagulation defects, polycythemia). Also includes hemorrhagic disorder due to circulating anticoagulants (HIT), fibrinolytic defects, non-thrombocytopenic purpura, thrombophilia, platelet defects, thrombocytosis, thrombocytopenia, thrombotic microangiopathy (World Health Organization, 2015). This category does not include all anticoagulated patients but includes patients with more than one DVT or other clot, has an inferior vena cava filter for clots, or other evidence of heightened risk of clotting or bleeding.
14. **Anemia documented** includes blood loss anemias, hemolytic, metabolic, aplastic, and nutrient deficiency anemias. Includes thalassemias and sickle cell disorders (World Health Organization, 2015). Includes patients on prescribed B12 injections because ICD-11 draft describes multiple types of Vitamin B12 deficiency anemia and pernicious anemia whereby Vitamin B12 injections would be the treatment (World Health Organization, 2015) (Use blood level as an alert, but anemia must be stated unless transfusion given with low Hgb).
15. **Fluid and electrolyte disorders documented** including volume depletion, fluid overload, hypernatremia, hyponatremia, hyperkalemia, hypokalemia, hyper or hypo magnesium or calcium. Also includes acidosis or alkalosis (World Health Organization, 2015). (Include if abnormal lab values are present AND treatment was provided (e.g. with potassium, or kaexylate, or magnesium, etc.) (Team concensus).
16. **Obesity -** Abnormal or excessive fat store secondary to different causes including energy imbalance, drugs, and genetic disorders. Includes any documentation of the words ‘obese or obesity’ (mild, moderate, or severe; morbidly obese). A condition characterized by excess weight relative to height. Overweight and obesity are assessed by the body mass index (BMI). The BMI is a measure of body mass relative to height, calculated as weight (kg)/height² (m²). For adults, overweight is defined by a BMI ranging from 25.00 to 29.99 kg/m² and obesity by a BMI greater than or equal to 30.00. There are three levels of severity in obese adults in recognition of different management options (World Health Organization, 2015). (Includes patients with BMI >30).
17. **Significant recent weight loss** is a symptom (not a condition) that accompanies other conditions; usually having occurred within the past 3-6 months. Includes cachexia, anorexia, or other specifically documented weight loss that may be related other conditions such as, but not exclusively, depression, cancer, or failure to thrive (Quan et al., 2005; World Health Organization, 2015).
18. **Dementia** includes patients with chronic cognitive deficit. Includes Alzheimer disease dementia, vascular dementia, Parkinson disease dementia, or dementias due to brain atrophy, infections, toxins, and metabolic abnormalities (World Health Organization, 2015).
19. **Human immunodeficiency virus disease/ Acquired immune deficiency syndrome** (HIV/AIDS). A case of HIV infection is defined as an individual with HIV infection irrespective of clinical stage including severe or stage 4 clinical disease, (also known as AIDS) confirmed by laboratory criteria. Includes patients with definite or probable AIDS, i.e. AIDS related complex (World Health Organization, 2015).
20. **Tobacco use (cigarette smoking)** includes current nicotine dependence with nicotine use within the past month and regular use of chewing tobacco (World Health Organization, 2015). Does not include nicotine patches, gum, or e-cigarettes.
    1. Current smoker (any documented amount of current smoking)
    2. Non-smoker (denies currently smoking; must be documented as non-smoker, even if recently quit smoking)
    3. No information
21. **Dyslipidemia** includes hyperlipidemia or hyperlipoproteinemia; disorders of lipoprotein metabolism and certain specified lipidaemias (World Health Organization, 2015). Include if the patient is on a lipid lowering drug.

**Statins**: Altocor®, Mevocor® (lovastatin), Crestor® (rosuvastatin), Lescol® (fluvastatin), Lipitor® (atorvastatin), Pravachol® (pravastatin), Zocor® (simvastatin), Baycol®

**Fibrates**: Antara®, Lipofen®, Tricor®, Triglide® (fenofibrate), Atromid-S® (clofibrate), Lopid® (gemfibrozil); Cholesterol Absorption Inhibitors: Zetia® (ezetimibe)

**Bile Acid Sequestrants:** Colestid® (colestipol), Questran® (cholestyramine), Welchol® (colesevelam); Nicotinic Acid Group: Niacor® (niacin)

**Fish Oil:** Lovaza®, Omacor® (omega-3-acid ethyl esters)

**Combination Drug Therapy**: Advicor® (niacin/lovastatin), Simcor® (niacin/simvastatin), Vytorin® (ezetimibe/simvastatin)

Lipid panel present? Y/N if yes, input the values from SCM labs first, then paper records:

- 1. Total cholesterol (TC) (mg/dL)
  2. High density lipoprotein cholesterol (HDL) (mg/dL)
  3. Low density lipoprotein cholesterol (LDL) (mg/dL)
  4. Triglycerides (mg/dL)
  5. Total to HDL ratio (TC/HDL) (calculated during analysis) (So et al., 2010; Southern et al., 2017).

1. **Disorders due to use of alcohol (alcohol abuse)** - are characterized by the pattern and consequences of alcohol use. Included are Alcohol intoxication, Harmful use of alcohol, Alcohol dependence, Alcohol withdrawal, and Alcohol-induced mental and behavioural disorders (specific types of mental or behavioural symptoms developing in the context of alcohol use) (World Health Organization, 2015).
2. **Disorders due to psychoactive drug use (drug abuse)** - Disorders Due to Substance Use are mental and behavioural disorders that develop as a result of the use of predominantly psychoactive substances, including medications. Disorders due to substance use include Substance intoxication, Harmful use of substances, Substance dependence, Substance withdrawal, and Substance-induced mental disorders (specific types of mental or behavioural symptoms developing as a result of substance use). Includes opioids, cannabinoids, sedatives, hypnotics, anxiolytics, cocaine, amphetamines, caffeine, hallucinogens, inhalants, ecstasy, PCP, or other psychoactive substances (World Health Organization, 2015).
3. **Psychoses** - Schizophrenia and other primary psychotic disorders are characterized by significant impairments in reality testing and alterations in behavior manifest in positive symptoms such as persistent delusions, persistent hallucinations, disorganized thinking (typically manifest as disorganized speech), grossly disorganized behavior, and experiences of passivity and control, negative symptoms such as blunted or flat affect and avolition, and psychomotor disturbances. The symptoms occur with sufficient frequency and intensity to deviate from expected cultural or subcultural norms. These symptoms are the primary features of these disorders; they do not arise as a feature of another mental and behavioural disorder (e.g., a mood disorder, delirium, or a disorder due to substance use) (World Health Organization, 2015). (Use antipsychotic meds as a cue to look for psychosis which must be stated, or clusters of the stated symptoms must be present).
4. **Anxiety -** documented as a symptom or diagnosis of a disorder. Anxiety and fear-related disorders are characterized by excessive fear and anxiety and related behavioural disturbances, with symptoms that are severe enough to result in significant distress or significant impairment in personal, family, social, educational, occupational, or other important areas of functioning. Anxiety disorders include generalized anxiety disorder, social anxiety disorder, separation anxiety disorder, panic disorder, agoraphobia, specific phobia, psycho-active drug-induced anxiety disorder (World Health Organization, 2015). Include if prescribed anti-anxiety meds at admission AND discharge OR statement of anxiety in one or more places in the chart. Some evidence of chronicity is preferred.

**Anti-anxiety medications:** Benzodiazepines Used to Treat Anxiety

·         Alprazolam (Xanax®)

·         Clonazepam (Klonopin®)

·         Diazepam (Valium®)

·         Lorazepam (Ativan®)

·         Oxazepam (Serax®)

·         Chlordiazepoxide (Librium®)

1. **Depression** - Depressive disorders are characterized by depressive mood (e.g., sad, irritable, empty) or loss of pleasure accompanied by other cognitive, behavioural, or neuro-vegetative symptoms that significantly affect the individual’s ability to function. A depressive disorder should not be diagnosed in individuals who have ever experienced a manic, mixed or hypomanic episode, which would indicate the presence of a bipolar disorder (World Health Organization, 2015). Include if prescribed antidepressant drugs at admission AND discharge OR have a clear diagnosis of depression.

**Selective serotonin reuptake inhibitors (SSRIs)**

- Fluoxetine (Prozac®)
- Fluvoxamine (Luvox®)
- Sertraline (Zoloft®)
- Paroxetine (Paxil®, Seroxat®)
- Escitalopram oxalate (Lexapro®, Cipralex®)
- Citalopram (Celexa)

**Serotonin and norepinephrine reuptake inhibitors (SNRIs)**

- Venlafaxine (Effexor®)
- Duloxetine (Cymbalta®)
- Desvenlafaxine (Pristiq®). (Note: These drugs are used to treat depression, anxiety problems and chronic pain, so look for depression diagnosis).

**Norepinephrine and dopamine reuptake inhibitors (NDRIs)**

- Bupropion (Wellbutrin®, Zyban®) Note: These drugs are also used to treat attention-deficit/hyperactivity disorder and as a smoking cessation aid. Look for depression diagnosis.

**Noradrenergic and specific serotonergic antidepressants**

- Mirtazapine (Remeron®)

**Cyclics**

- Amitriptyline (Elavil®)
- Maprotiline (Ludiomil®)
- Imipramine (Tofranil®)
- Desipramine (Norpramin)
- Nortriptyline (Novo-Nortriptyline®)
- Clomipramine (Anafranil®)

**Monamine oxidase inhibiotrs (MAOIs)**

- Phenelzine (Nardil®)
- Tranylcypromine (Parnate®)
- Moclobemide (Manerix®)

1. **Homeless** – documented lack of housing, problem of homelessness, lack of shelter, social migrant, tramp, transient, or vagabond (World Health Organization, 2015).

**INFECTIONS**

Was **any infection** diagnosed during this admission? Y/N

1. **Urinary tract infection** includes a condition of the lower or upper urinary tract, caused by an infection with a bacterial source. This condition is characterized by loin tenderness, renal mass, large prostate, meatal ulcers, vaginal discharge, hypertension, or signs of chronic renal failure. Confirmation is by identification of the infectious agent (World Health Organization, 2015). (Include if urinalysis AND C&S are positive AND treatment is present- team consensus).
2. **Pneumonia** includes a disease of the lungs, frequently but not always caused by an infection with bacteria, virus, fungus, or parasite. This disease is characterized by fever, chills, and cough with sputum production, chest pain and shortness of breath. Confirmation is by chest x-ray (World Health Organization, 2015).
3. **Skin infection/ wound infection** - Infections and infestations affecting the skin incorporate both direct invasion of the skin (including associated mucous membranes, hair and nails) by microorganisms or parasites and dermatoses arising from systemic or other distant infections (e.g. viral exanthems). Includes entire skin and subcutaneous tissue (World Health Organization, 2015). (Includes Thrush in mouth, and vaginal yeast/candida or yeast at other skin locations; also includes MRSA+ unless specifically documented from another site - team consensus).
4. **GI infection / Gastroenteritis** includes any condition of the intestines, caused by an infection with a bacterial, viral, fungal, or parasitic source (World Health Organization, 2015). (Includes VRE+)
5. **Other** – e.g. tonsillitis, strep throat, influenzas, sexually transmitted diseases, hepatitis (World Health Organization, 2015).
6. **Sepsis** -This is a potentially deadly medical condition characterized by a whole-body inflammatory state (called a systemic inflammatory response syndrome or SIRS) caused by severe infection. Includes severe sepsis defined as sepsis plus sepsis-induced organ dysfunction or tissue hypo-perfusion (World Health Organization, 2015).

**PRESSURE ULCER**

1. **Pressure (decubitus) ulcer** – **Were any pressure ulcers documented?** Y/N Includes ulcers resulting from localized injury and ischemic necrosis of skin and underlying tissues due to prolonged pressure, or pressure in combination with shear; bony prominences of the body are the most frequently affected sites; immobility and debility are major contributing factors. Synonyms include bedsore, pressure injury, pressure sore, pressure necrosis of the skin (World Health Organization, 2015). Related words include stage, ulcer, wound, sore, reddened, open, coccyx, sacrum (Enterostomal nurse, JR, RN).

**Do one or more pressure ulcers appear in the discharge summary list of conditions/problems**? Y/N

**If yes, which documents contained the info (check all that apply)**

- 1. Emergency department notes
  2. History & Physical
  3. Multidisciplinary Progress Note
  4. Patient Assessment Flowsheet
  5. Patient Assessment - Neuro (Wound)
  6. Enterostomal Therapist RN note
  7. Specialist consultation note
  8. Allied health note (OT, PT)
  9. Orders
  10. Other - please describe _________

Pressure ulcer **locations** can include:

- 1. Ankle; b) Elbow; c) Foot; d) Heel; e) Ischial tuberosity; f) Leg; g) Occiput; h) Sacral/ coccyx; i) Trochanteric/ hip; j) other

**Pressure ulcer stage/severity** – see list- note only if specifically documented

**Staging documented by whom?**

2^nd^ to 3^rd^ pressure ulcer –questions repeated

**SLEEP DISORDERS**

Were **any sleep disorders documented**? Y/N (Use sleep aid meds as a cue to look for sleep disorders. Team consensus).

1. **Insomnia** - Disorders of initiating and maintaining sleep. Can be short-term or chronic. Chronic insomnia is defined as A condition of unsatisfactory quantity and/or quality of sleep, which persists for a considerable period of time, including difficulty falling asleep, difficulty staying asleep, or early final wakening. Insomnia is a common symptom of many mental and physical disorders and should be classified here in addition to the basic disorder only if it dominates the clinical picture. Also called nonorganic origin insomnia, sleep disorder, or hyposomnia (World Health Organization, 2015). (Include if hypnotics prescribed at admission AND discharge or explicit statement of insomnia- team consensus).

**Hypnotics:**

- Eszopiclone (Lunesta®)
- Ramelteon (Rozerem®)
- Zolpidem (Sublinox®)
- Zopiclone (Imovane®)
- Zaleplon (Sonata®)

1. **Hypersomnia** - Disorders of excessive somnolence (excluding narcolepsy or cataplexy). Includes primary hypersomnia, hypersomnia due to other mental disorder, idiopathic hypersomnia, recurrent hypersomnia, hypersomnia due to medical condition (World Health Organization, 2015).
2. **Disorders of the sleep- wake schedule** (also called circadian rhythm sleep disorders)(1), includes unspecified, delayed sleep phase type, advanced sleep phase type, irregular sleep wake type, free running type, jet lag type, shift work type, or other (Ingelsson et al., 2007).
3. **Sleep apneas** include one or combinations of the following (may choose more than one). Combinations may be referred to as complex sleep apnea, mixed obstructive and central sleep apnea, or CPAP emergent central apnea (sleep specialist physicians WT, SP).
   1. **Central sleep apnea** which includes Cheyne-Stokes respiration; central sleep apnea secondary to <insert disea.se/drug>
   2. **Obstructive sleep apnea** (OSA); OSA with hypoxemia; OSA with hypoventilation
   3. **Sleep-related hypoventilation;** hypoxemia disorders related to sleep; obesity hypoventilation syndrome (World Health Organization, 2015).
4. **Narcolepsy** **with or without** **cataplexy** - Narcolepsy with cataplexy is a sleep disorder characterized by excessive day-time sleepiness associated with uncontrollable sleep urges and cataplexy (loss of muscle tone often triggered by pleasant emotions). Narcolepsy without cataplexy is characterized by excessive day-time sleepiness associated with uncontrollable sleep urges and sometimes paralysis at sleep, hypnagogic hallucinations and automatic behavior (World Health Organization, 2015).
5. **Restless legs syndrome** refers to an unpleasant crawling sensation in the legs or arms, particularly when sitting and relaxing in the evening, that improve upon walking. Akathisia is an inability to sit still. There are two aspects to akathisia: a subjective report of restlessness or inner tension, particularly referable to the legs, with a consequent inability to maintain a posture for several minutes, and the objective (or observational) manifestations of restlessness in the form of semi-purposeful or purposeless movements of the limbs, a tendency to shift body position in the chair while sitting, or marching on the spot while standing etc. (World Health Organization, 2015).

**Notes about this patient** – include primary admitting diagnosis if not already identified (e.g. failure to thrive, fractured femur).

**HARMS / ADVERSE EVENT**

1. **Did this patient experience a harm (injury, illness, disability at the time of discharge, prolonged hospital stays, or death) from an adverse event? Yes or No**
   1. Adverse events are unintended injuries or complications that are **caused** by health care management, rather than by the patient's underlying disease and they lead to harm (de Silva, 2014). Include events that shouldn’t happen (e.g. UTI, fall, ventilator acquired pneumonia), even if unable to determine a healthcare related cause or mode (Team consensus).
2. **If yes, harm type**
   1. Hospital-acquired infection
   2. Decubitus (pressure) ulcer
   3. Endocrine or metabolic complication
   4. Venous thromboembolic event
   5. Cardiac complication
   6. Respiratory complication
   7. Hemorrhagic event
   8. Drug related adverse event
   9. Adverse events related to fluid management
   10. Complications directly related to surgery
   11. Traumatic injuries (non-procedural) arising in hospital
   12. Anesthesia related complications
   13. Delirium
   14. Central nervous system complications
   15. Gastrointestinal
   16. Severe life or major vital organ threatening event
3. **What is the cause of the harm?**
   1. Drug or biological substance
   2. Device
   3. Procedure
   4. Other aspects of care associated with injury or harm
   5. Unable to determine
4. **What is the cause? Specify drug or biological substance associated with injury or harm.**
   1. Systemic antibiotics
   2. Systemic anti-infectives and antiparasitics
   3. Hormones
   4. Primary systemic agents
   5. Agents primarily affecting blood constituents
   6. Analgesics, antipyretics and anti-inflammatory drugs
   7. Antiepileptics and antiparkinsonism drugs
   8. Sedatives, hypnotics and antianxiety drugs
   9. Psychotropic drugs, not elsewhere classified
   10. Central nervous system stimulants, not elsewhere classified
   11. Drugs primarily affecting the autonomic nervous system
   12. Drugs primarily affecting the cardiovascular system
   13. Drugs primarily affecting the gastrointestinal system
   14. Drugs primarily affecting water-balance and mineral and uric acid metabolism
   15. Drugs primarily acting on smooth and skeletal muscles and the respiratory system
   16. Topical agents primarily affecting skin and mucous membrane and ophthalmological, otorhinolaryngological and dental drugs
   17. Other and unspecified drugs and medicaments
   18. Bacterial vaccines
   19. Other and unspecified vaccines and biological substances
   20. Complementary and Traditional Medicines
   21. Other specified drugs medicaments and biological substances associated with injury or harm in therapeutic use
   22. Drugs medicaments and biological substances associated with injury or harm in therapeutic use, unspecified
5. What is the cause? Specify surgical or medical devices associated with injury or harm
   1. Anaesthesiology devices
   2. Cardiovascular devices
   3. Otorhinolaryngological devices
   4. Gastroenterology and urology devices
   5. General hospital and personal use devices
   6. Neurological devices
   7. Obstetric and gynaecological devices
   8. Ophthalmic devices
   9. Radiological devices
   10. Orthopaedic devices
   11. Physical medicine devices
   12. General and plastic surgery devices
   13. Other and unspecified medical device, describe
6. What is the cause? Specify surgical or medical procedures associated with injury or harm
   1. Neurological procedure
   2. Cardiac procedure
   3. Thoracic procedure
   4. Gastrointestinal procedure
   5. Endocrine procedure
   6. Gynaecological procedure
   7. Urological procedure
   8. Obstetric procedure
   9. Orthopaedic procedure
   10. Vascular procedure
   11. Ear, nose and throat procedure
   12. Dental procedure
   13. Plastic surgery procedure
   14. Other specified procedure
   15. Unspecified procedure
   16. Other specified medical or surgical procedure associated with injury or harm in therapeutic use
   17. Medical or surgical procedure associated with injury or harm in therapeutic use, unspecified
7. What is the cause? Specify which other aspects of care were associated with injury or harm?
   1. Non-administration of necessary drug
   2. Non provision of necessary procedure
   3. Problem associated with physical transfer of patient
   4. Mismatched blood used in transfusion
   5. Other problem associated with transfusion
   6. Problem associated with physical restraints
   7. Problem associated with isolation of patient
   8. Problem associated with clinical documentation
   9. Problem associated with clinical software
   10. Problems associated with diagnosis
   11. Problem associated with delayed treatment
   12. Problem associated with transitions of care, hand offs, or handovers
   13. Other specified other aspects of care associated with injury or harm
   14. Other aspects of care associated with injury or harm, unspecified
8. Other cause of harm not listed? Please specify.
9. If a harm from an adverse event took place, was the CAUSE clearly documented?
   1. Not clear/ absent to very clear
   2. Clearly documented means easy to find, made sense, thorough
10. Mode of harm
    1. Mode of injury or harm associated with exposure to a drug, medicament or biological substance
    2. Mode of injury or harm associated with a surgical or other medical device
    3. Mode of injury or harm associated with a surgical or other medical procedure
    4. Conditions associated with medical facility or provider performance
    5. Unable to determine
11. Specify how the mode of injury or harm was associated with a drug or biological substance
    1. Overdose of substance
    2. Underdosing
    3. Drug-related injury or harm in context of correct administration and dosage
    4. Unspecified appropriateness of dosing or administration
    5. Incorrect duration of administration or course of therapy
12. Specify how the mode of injury or harm was associated with a surgical or medical device
    1. Structural device failure
    2. Functional device failure
    3. Operator error
    4. Combination or integration of operator error and device failure
    5. Unintentional dislodgement, misconnection or de-attachment
    6. Infection of device
    7. Obstruction of device
13. Specify how the mode of injury or harm was associated with a surgical or medical procedure
    1. Cut or puncture, as mode of injury
    2. Perforation, as mode of injury
    3. Burn arising during procedure, as mode of injury
    4. Embolisation, as mode of injury
    5. Foreign body accidentally left in body, as mode of injury
    6. Failure of sterile precautions
    7. Procedure undertaken at wrong site or wrong side, as mode of injury
    8. Pressure, as mode of injury
    9. Other specified mode of injury or harm associated with a surgical or other medical procedure
    10. Mode of injury or harm associated with a surgical or other medical procedure, unspecified
14. Specify how the mode of injury was related to conditions associated with medical facility or provider performance
    1. Provider performance compromised by insufficient training
    2. Provider performance compromised by insufficient or inadequate supervision
    3. Provider performance compromised by sleep deprivation
    4. Provider performance compromised by excessive workload
    5. Provider performance compromised by substance use or abuse
    6. Failure to connect with or identify the need for various needed services provided by medical facility
    7. Insufficient medical facility staffing
    8. Unavailability and inaccessibility of health care facilities
    9. Person awaiting admission to adequate facility elsewhere
    10. Medical services not available in current medical facility
    11. Medical services not available in home
    12. Respite care
    13. Other specified factors associated with health facility or provider performance
    14. Factors associated with health facility or provider performance, unspecified
    15. Concern about or fear of medical treatment
15. Other mode of harm not listed. Please specify.
16. If a harm from an adverse event took place, was the MODE clearly documented?
    1. Not clear/ absent to very clear
    2. Clearly documented means easy to find, made sense, thorough
17. Did this patient experience a 2^nd^ harm? Content repeats for 2^nd^ to 5^th^ harms.

**REFERENCES**

de Silva D (2014) Helping measure. (March 2014): 80.

Ingelsson E, Keyes MJ, Pencina MJ, et al. (2007) Clinical utility of different lipid measures for prediction of coronary heart disease in men and women. *Journal of the American Medical Association* 298(7): 776–785. DOI: 10.1001/jama.298.7.776.

Quan H, Sundararajan V, Halfon P, et al. (2005) Coding algorithms for defining comorbidities in ICD-9-CM and ICD-10 administrative data. *Medical care* 43(11). United States: 1130–1139.

So L, Beck CA, Brien S, et al. (2010) Chart documentation quality and its relationship to the validity of administrative data discharge records. *Health Informatics Journal* 16(2): 101–113. DOI: 10.1177/1460458210364784.

Southern DA, Burnand B, Droesler SE, et al. (2017) Deriving ICD-10 Codes for Patient Safety Indicators for Large-scale Surveillance Using Administrative Hospital Data. *Medical Care* 55(3): 252–260. DOI: 10.1097/MLR.0000000000000649.

World Health Organization (2015) ICD-11 Beta Draft (Joint Linearization for Mortality and Morbidity Statistics. Available at: http://apps.who.int/classifications/icd11/browse/l-m/en - /http%3A%2F%2Fid.who.int%2Ficd%2Fentity%2F1703442464.

World Health Organization (2016) International Statistical Classification of Diseases and Related Health Problems 10th Revision (ICD-10)-WHO Version. Available at: https://icd.who.int/browse11/l-m/en#/http://id.who.int/icd/entity/1890228613.

**ACKNOWLEDMENTS**

We would like to thank the chart reviewers: Olga Grosu, Chris King, Natalie Wiebe, Ellena Kim, Danielle Fox, and Nicholas Van Kampen, and the authors for their valuable input during early development of definitions and inter-rater reliability testing phase.
