# Supplementary material for: Field testing a new ICD coding system: methods and early experiences with ICD-11 Beta Version 2018
Source: BMC Res Notes. 2022 Nov 8;15:343. doi: 10.1186/s13104-022-06238-2 (PMC9644463; doi:10.1186/s13104-022-06238-2)
Supplement: Supplementary file 3 — Additional file 3. Comparison between ICD-10-CA and ICD-11 Coding Structure [file 13104_2022_6238_MOESM3_ESM.docx]

**Additional File 3 Comparison between ICD-10-CA and ICD-11 Coding Structure**

| **ICD-10-CA Coding structure** | **ICD-11 Coding Structure** |
| --- | --- |
| **Example 1**  **Dxcode1 dxtype1 Dxcode2 dxtype2 Dxcode3 dxtype3**  I21.0 M R94.30 3 I25.10 1  Description: (3 fields listed separately and **not** flagged with a cluster to note relationship).   - I21.0: Acute transmural myocardial infarction of anterior wall (Main condition (dxtype=M)) - R94.30: Electrocardiogram suggestive of ST segment elevation myocardial infarction [STEMI] (secondary diagnosis (dxtype=3)) - I25.10: Atherosclerotic heart disease of native coronary artery (pre-admit comorbidity (dxtype=1)) | **Dxcode1**  BA41.0&XA7RE3/BA80.0&XA7NQ7&XY6E&XY7B&XY6M  Description: (single field with all codes listed together to note relationship)   - *Stem code:* Acute ST elevation myocardial infarction (BA41.0) & Anterior wall of heart (XA7RE3) - Coronary atherosclerosis of native coronary artery (BA80.0) & Left anterior descending coronary artery (XA7NQ7) - *Discharge Diagnosis Type:* Initial Reason for Admission (XY6E), Main Resource (XY7B)   *Timing information:* Present on Admission (XY6M) |
| **Example 2**  **Dxcode1 dxtype1 Dxcode2 dxtype2 Dxcode3 dxtype3**  S52000 M W00 9 U98.9 9  **Description:** (3 fields listed separately and **not** flagged with a cluster to note relationship).   - S52.000: Fracture of olecranon process of ulna, closed (Main Condition (dxtype=M)) - W00: Fall on same level involving ice and snow (S52.000 codes requires external cause of injury code (dxtype 9) to be coded) - U98.9: Unspecified place of occurrence S52.000 codes requires place of occurence code (dxtype 9) to be coded) | **Dxcode1**  NC32.0&XK9K&XJ8PQ&XJ44E&XA5VA1/PA60&XE3LV&XE53A  &XY6E&XY7B&XY6M    **Description:** (single field with all codes listed together to note relationship)   - *Stem code:* Fracture of upper end of ulna (NC32.0) & Right (XK9K) & Displaced fracture (XJ8PQ) & Closed fracture (XJ44E) & Olecranon process of the ulna (XA5VA1) - *Information about external cause of injury:* Unintentional fall on the same level or from less than 1 metre (PA60) & Snow, ice (XE3LV) & sidewalk (XE53A) - *Discharge Diagnosis Type:* Initial Reason for Admission (XY6E), Main Resource (XY7B)   *Timing information:* Present on Admission (XY6M) |
| **Example 3**  **Dxcode1 Dxtype1 dxcluster1 dxcode2 Dxtype2 dxcluster2**  T81.0 M A T81.1 1 A  **Dxcode3 Dxtype3 Dxcluster3 Dxcode4 Dxtype4 Dxcluster4**  T81.2 1 A S36.510 3 A  **Dxcode5 Dxtype5 Dxcluster5**  Y60.0 9 A  **Description:** (5 fields listed separately but flagged with a cluster to note relationship)   - T81.0: Haemorrhage and haematoma complicating a procedure, not elsewhere classified (Main condition (dxtype=M)) - T81.1: Shock during or resulting from a procedure, not elsewhere classified (pre-admit comorbidity (dxtype=1)) - T81.2: Accidental puncture and laceration during a procedure, not elsewhere classified (pre-admit comorbidity (dxtype=1)) - S36.510: Laceration of colon, without open wound into cavity (secondary diagnosis (dxtype=3)) - Y60.0: During surgical operation (S36.510 requires external cause of injury code (dxtype=9)) | **Dxcode1**  NE81.0Y/PK80.32/PL11.Z&XY6E&XY7B&XY0Y          **Description:** (single field with all codes listed together to note relationship)   - *Stem code:* Haemorrhage or haematoma of other specified site complicating a procedure, not elsewhere classified (NE81.0Y) - *Information regarding cause of harm:* Gastrointestinal, abdominal, or abdominal wall procedure associated with injury or harm, endoscopic approach (PK80.32) - *Information regarding mode of harm:* Unspecified mode of injury or harm associated with a surgical or other medical procedure (PL11.Z) - *Discharge Diagnosis Type:* Initial Reason for Admission (XY6E), Main Resource, Main Condition (XY7B) - *Timing information:* Present on Admission (XY0Y) |
