# Supplementary material for: Field testing a new ICD coding system: methods and early experiences with ICD-11 Beta Version 2018
Source: BMC Res Notes. 2022 Nov 8;15:343. doi: 10.1186/s13104-022-06238-2 (PMC9644463; doi:10.1186/s13104-022-06238-2)
Supplement: Supplementary file 4 — Additional file 4. Examples of changes made to ICD-11 [file 13104_2022_6238_MOESM4_ESM.docx]

**Additional file 4 Examples of changes made to ICD-11**

| **Category** | **Clinical Coder Comment** | **Examples** | **Action taken by the WHO consultant and/or a WHO advisory group** |
| --- | --- | --- | --- |
| Post-coordination | No option/ unavailable codes | FA01.0 Primary osteoarthritis of knee missing post-coordination for laterality and specific anatomy | Laterality, specific anatomy, and has manifestation of… were added to the browser for all types of osteoarthritis |
|  | The anatomy and the histopathology are already specified in the code; redundant to add the cervix uteri extension code | 2D27.0 Squamous cell carcinoma of cervix uteri | Post coordination of anatomy has been removed |
|  | No laterality in post coordination | FA2Z Inflammatory arthropathies, unspecified | Added to browser |
|  | Code is post-coordinated with itself. | GA90 Hyperplasia of prostate. The post-coordination option directed to the code GA90 itself causing a loop. | Post coordination has been removed |
|  | Missing specific anatomy. | Other toe fracture codes available. Fracture of great toe lacked a specific code. | Corrected in the browser to become ND13.4 Fracture of great toe. |
|  | Missing extension codes | There was no extension code for Enterococcus casseliflavus  Only XN1F7 Enterococcus was present | XN3XY Enterococcus casseliflavus was added to the browser |
| Documenta-tion | Detailed codes are available but there is a lack of documentation. For example, it was difficult to code some psychiatric conditions. The documentation rarely supported specific codes given all the details in the psychiatric codes. | Anorexia nervosa with significantly low or dangerously low body weight were the choices. | Feedback given to Mental Health Technical Advisory Group  6B80.Z Anorexia Nervosa, unspecified has since been added to the browser after response from Mental Health Technical Advisory Group |
| Missing Codes/ Inclusion Terms | Missing codes for certain conditions | XA3TG4 Intestinal Lymph nodes | Anatomy description codes added to the browser |
|  | Duplicate codes | Two extension codes for shoulder joint: XA05J7 Shoulder joint and XA49P8 Glenohumeral joint | Duplicate codes were removed from browser |
|  | Require code for inclusion term as an addition to the cluster | DC11.0 Calculus of gallbladder or cystic duct with acute cholecystitis: inclusion term- Gangrene of gallbladder with calculus | Was under discussion to be added. Gangrene of gallbladder with calculus not added as inclusion term. |
|  | Caregiver burnout - could not find a good fit. In ICD 10 the code goes to Z74.2Need for assistance at home and no other household member able to render care, and I could not find this | No code for caregiver burnout | New code added: QF27 Difficulty or need for assistance at home and no other household member able to render care |
| Substance/ Medication List | No codes for specific meds | Benzodiazepine results under two different categories: Central nervous system depressant benzodiazepine and tranquilizer benzodiazepine | List has been rebuilt |
| Harms (3- part model) | Unable to find code | Titanium metalosis as a harm/injury due to dislocation of cemented polyethylene liner | Proposal was initiated to classify code to **NE83.Y** Other specified injury or harm arising from other device, implant, or graft, not elsewhere classified. Change now appears in the browser. |
|  | Infection codes required specificity | Infection codes needed – as it could be either local (insertion site) or sepsis.  PH71.15 Cardiovascular devices associated with adverse incidents: central venous catheter  PH91.5 Infection associated with device, implant, or graft as mode of injury – is the 3-part model for line infections | Topic was referred to Quality and Safety Technical Advisory Group and Morbidity Reference Group  Reference guide content was updated for clarity related to sepsis and central line infection coding*. New code include:  (Harm) NE83.1 Infection arising from other device, implant, or graft, not elsewhere classified.  (Cause) PK91.15 Cardiovascular devices associated with injury or harm, central venous catheter.  (Mode) Unless there is mention of failure of sterile precautions, code PL12.Z Mode of injury or harm associated with a surgical or other medical device, implant, or graft, unspecified. |
|  | No option to post-coordinate the mode | 4B00.01 Acquired neutropenia - there is the option to post coordinate the cause but not mode. | Topic referred to Quality and Safety Technical Advisory Group to clarify if the 3-part model applies. Detailed content was added to the reference guide for applying the 3-part model*. |
|  | Unclear when to use the 3-part model for coding healthcare related harms | Overdose vs. poisoning. Adult male receives an overdose of prescribed medication when an excess dose was inadvertently injected by a nurse in the hospital. | Ten examples and a flowchart were drafted and are since adopted into the ICD-11 Reference Guide (2.25.5) |
| Coding tool | Unable to find code | 6C4G.1Z polysubstance abuse | Added to browser |

*3-part model refers to harm/injury, cause, mode. See ICD-11 Reference Guide section 2.24.17 Conceptual model for quality and patient safety.
